# A Radiometric Study of Factors Affecting Drug Output of Jet Nebulizers

**DOI:** 10.4103/0250-474X.62234

**Published:** 2010

**Authors:** G. Mittal, N. Kumar, H. Rawat, M. K. Chopra, A. Bhatnagar

**Affiliations:** Institute of Nuclear Medicine and Allied Sciences, Defence R & D organization, Brig. S. K. Mazumdar Road, Delhi-110 054, India

**Keywords:** Jet nebulizer performance, radiometry, Technetium-99m

## Abstract

Jet nebulizers show an unreasonable variation in drug output and nebulization rates that leads to clinical and regulatory problems. Current evaluation methods appear inadequate for the purpose. Our objective was to evaluate Technetium-99m radiometry to study nebulizer parameters and the factors influencing it quantitatively. Drug output, output rate and residual mass and the effect of excipient, temperature, surface tension, air-jet speed, and equipment brand and aging were studied. Though nebulization of radiolabeled drugs followed first-order kinetics, the rates were significantly different; the heaviest drug (Tc-99m colloid) and Tc-99m salbutamol had the least nebulization. Nebulization rate for the first minute was invariably higher than the mean rate signifying the concentration effect of the solute. Drug residue was 35-75%. Drug output of different nebulizer chamber and air compressor brands was different to the extent of 270% and 180% respectively. ‘Aging’ of fluid chamber, cold drug fluid and obstruction in air-jet resulted in significant reduction in output, while addition of 2% saline as excipient did not change the output rate. Addition of ethyl alcohol resulted in a maximum of 260% enhancement (with Tc-99m salbutamol), while further reduction in surface tension was counterproductive irrespective of the drug used. We conclude that radiometry can provide valuable parametric information on the performance of different jet nebulizers.

Medical nebulizer is one of the most commonly used biomedical devices. It aims to deliver a therapeutic dose of the drug as an aerosol within a short period of time, usually 5-10 min. The commonest indications are delivery of bronchodilators and steroids in asthma and other obstructive airway diseases though it is also used to deliver antimicrobials and in palliative care[1,3]. With inhalation becoming a preferred route for delivering a number of drugs for systemic action, the utility spectrum of nebulizer system is poised for a higher degree of use. Inhalation is now becoming the preferred route for a number of drugs for instant systemic delivery, including insulin and other hormones, painkillers and antibiotics[4–6].

It is a matter of concern that more than a century after its invention, measurement of drug delivery through the nebulizer remains a difficult and underdeveloped science[7]. There is an unreasonable variation in nebulised dosage and by implication unreasonable differences in the clinical effects[8,9]. Output of a given drug from different nebulizers may vary up to 400% and output of a given nebulizer may vary dramatically depending on the drug nebulized[10–13]. Studies on bronchodilatory response and intrapulmonary deposition have shown marked differences, presumably representing variations in nebulization rate, output and droplet size[14–16]. Inhalation therapy will therefore be unfeasible with drugs that have a narrow therapeutic-safety ratio. Precise information about drug output and dose delivered has or is poised to become a mandatory regulatory requirement[7,17,18].

Although methods for estimating the total amount of drug reaching the lungs using either gamma scintigraphy[19,20] or pharmacokinetic measurements[21] exist, it is however not easy to characterize the nebulizer performance, more so since a variety of factors like respiratory particle fraction, their internal deposition pattern and drug mass output are known to influence nebulization rate and the present methods are inadequate to verify and quantify their influence[9,16]. Aerosol output has been used as an indirect measure of drug output using gravimetric method[22], but is highly inaccurate for jet nebulizers as it assumes static drug concentration in the fluid, which is incorrect. A modified method, factoring in the concentration of the drug in the residual fluid has also been found to overestimate drug delivery[23]. Yet another method assesses drug output by collecting the aerosol in a filter and analyzing its weight. A variant of this method is to substitute the drug by a chemical tracer provided it behaves like the parent drug[24,25]. For common bronchodilators, output data derived from 0.9% sodium chloride analysis has been used as a substitute[26]. However this will not be possible with other drugs like steroids and antibiotics that have physicochemical properties quite different from 0.9% sodium chloride[27]. The methods available presently for measuring nebulised drug output appear inadequate, more so because a variety of factors are known to influence nebulization rate but the present methods are inadequate to verify and quantify their influence. It is therefore important both from medical and regulatory points of view, that performance of each nebulizer-compressor system be specified and such analysis methods should be capable of measuring the influence of various given factors on the nebulizer drug output. Such methods need to be ubiquitous, accurate and simple to perform, considering the present and future expansion of the use of inhalation route of drug delivery.

Gamma scintigraphy, particularly with Tc-99m based radiopharmaceuticals, is considered the gold-standard method to estimate deposition pattern in human subjects[28,29]. It is believed that the technique can also be used with advantage for quantifying nebulizer performance and for studying the factors influencing it. Instruments quantifying the radioactivity show a linear response in low dose rates, a fact also used in our study. A number of radiopharmaceuticals are available that may share chemical similarity with different drugs. Salbutamol and other beta-1 agonists have been radiolabeled with Tc-99m and found to behave similar to the parent molecule *in vitro* as well as in animals and humans[30].

This communication describes use of Tc-99m based radiometry technique to provide reproducible information on drug output of nebulizers, and quantification of the effect of factors influencing drug output. Our purpose is not to characterize any particular compressor-nebulizer system with respect to output of any particular drug; it is limited to present the technical feasibility of this new application of radioisotopes.

## MATERIALS AND METHODS

A standard dose calibrator (Capintec, NJ, USA) set at measuring Tc-99m was used to measure radioactivity. Tc-99m pertechnetate was extracted using solvent-extraction method from a molybdenum generator. All other chemicals were of analytical grade procured from commercial sources. Triple distilled water was used for solution preparation. Unless specified, the experiments were conducted at room temperature (20-28°) using fresh nebulizer chambers from the same batch and a single medical air compressor (American Bantex Corp., Alphaneb Plus Nebulizer, NJ, USA). Tc-99m salbutamol sulphate was made by stannous chloride reduction method already described from our lab[30]. All other radiopharmaceuticals used were of high radiochemical and chemical purity made from human grade ‘cold’ kits.

### Study design:

The study was designed to quantify drug output and factors influencing it using Tc-99m based radiopharmaceuticals. Table 1 enlists the various tests performed in a modular fashion. Tc-99m pertechnetate (99mTcO_4_^−^) is chemically similar to sodium chloride, while other radiopharmaceuticals listed in Table 1 represent different types of organic molecules. Tc-99m sulphur colloid represents nebulisable colloids and suspensions. Tc-99m salbutamol sulphate was used in most experiments because it represents one of the most commonly nebulised drug[31].

**TABLE 1:** RADIOPHARMACEUTICALS USED AND TESTS PERFORMED

Radiopharmaceuticals	Tests performed
Tc-99m Pertechnetate	Mean and first minute nebulization rate
Tc-99m Sulphur Colloid	Drug output, Residual waste, Nature of drug
Tc-99m DTPA	Influence of fluid volume & drug concentration
Tc-99m Mebrofenin	Effect of excipients
Tc-99m MDP	Effect of driving gas flow, compressor make
Tc-99m Tetraphosphine	Effect of nebulizer make and chamber aging
Tc-99m MIBI	Effect of surface tension and temperature
Tc-99m Phytate	
Tc-99m Salbutamol	

### Method of nebulization and safety precautions:

The nebulization chamber and dose calibrator were housed behind lead shielding in fumehood. A small amount of radioactivity (4-6 drops of solution containing 3.7-12 MBq of a radiopharmaceutical) was placed in the nebulization chamber fluid. Following initiation of nebulization, the radio-aerosols were allowed to pass through the vent of the fumehood. The amount of aerosolized radioactivity was determined by measuring the remaining activity in the chamber. All experiments were performed following ALARA (as low as reasonably achievable) principle, and were approved by Institute's radiation safety officer.

### Estimation of drug output, output rates and residual mass of drug with different drugs:

Nebulizer chamber was filled with 3 ml triple distilled water, in which 3-4 drops of radiolabeled drug was added. In one set of experiments (n=6), original radioactivity and radioactivity remaining in the chamber was noted sequentially after every minute till end of nebulization. Total drug output, mean output rate and residual activity were calculated (Table 2). In another set of experiment the nebulization rate for radiopharmaceuticals was calculated for the 1^st^ minute of nebulization with constant volume of 3 ml (Table 3).

**TABLE 2:** DRUG OUTPUT PERFORMANCE OF A NEBULIZER

Drug	Drug output (%)	Residual mass %	Time of nebulization	Mean Rate nebulization (%min)
Tc-99m Salbutamol	36.1±4.2	63.9	22 min, 20 sec	1.6
Tc-99m DTPA	50.0±6.7	50.0	13 min 23 sec	3.7
Sodium Pertechnetate	27.8±6.1	73.2	11 min	2.5
Tc-99m GHA	61.5±12.8	38.5	12 min 19 sec	5.0
Tc-99m MIBI	59.5±14.0	40.5	14 min	4.3

Drug output performance of a nebulizer with respect to radiolabeled (Tc-99m) pharmaceuticals in 3 ml of distilled water (n=6) (mean ± S.D.). Mean rate of nebulization was obtained by dividing total output by total time of nebulization.

**TABLE 3:** COMPARISON OF DRUG OUTPUT RATES DURING FIRST MINUTE

Drug	Output rate/min (%)
Tc-99m Sulphur Colloid	2.10±1.8
Tc-99m Pertechnetate	4.25±2.5
Tc-99m DTPA	6.20±2.9
Tc-99m Mebrofenin	4.20±1.8
Tc-99m MDP	1.83±1.1
Tc-99m Tetraphosmine	3.20±1.8
Tc-99m MIBI	4.90±2.1
Tc-99m Phytate	3.10±0.9
Tc-99m Salbutamol	1.90±0.9
Tc-99m GHA	5.20±2.4

Drug output rate comparison during first minute of nebulization with fixed volume (3 ml) in distilled water (n=30 each)

### Effect of air compressor on drug output:

Radioactive fluid was nebulized using medical air compressors of two standard makes (air compressor 1 and 2) using the same nebulizer chamber and radiopharmaceutical preparations in 3 ml volume each. The air compressors have been given arbitrary code names since the present study only aims to highlight the utility of radiometry as a technique for assessing performance of jet nebulizers and not to create any commercial bias regarding different medical air compressors available in the market at this stage. Nebulization was done for 10 min and chamber radioactivity was noted every minute.

### Effect of air jet flow on drug output:

A small ball of cotton was placed in the tubing connecting output portal of the air-compressor with the air input portal of the nebulizer chamber. This was done to simulate obstruction caused by dried out salt or drug crystals in the air pathway. No attempt was made to determine the actual airflow rate. Nebulization was done with a volume of 3 ml for 10 min. Radioactivity outflow data were collected before and after airflow obstruction three times with different radiopharmaceuticals (n=30).

### Effect of excipient:

Aliquots of 3 ml radioactivity in water, 0.45, 0.9 and 2% saline as solvents were nebulised with same compressor-nebulizer system. Nebulization was done with a volume of 3 ml for 10 min. Radioactivity outflow data were collected before and after nebulization. The experiment was repeated three times with each radiopharmaceutical (n=30).

### Effect of surface tension:

Ethyl alcohol and Tween-80 were used for lowering surface tension of distilled water. No attempt was made to find the actual surface tension experimentally. Incremental concentration of the agents was used (5, 10, 20 and 30% ethyl alcohol and 0.5, 5 and 20% Tween 80 containing few MBq of radiopharmaceutical). Nebulization rate for the first minute was noted for each experiment (n=30). The experiment was repeated for different radiopharmaceuticals.

### Effect of solution temperature:

Ice-cold (4°) and hot water (50°) respectively was used to make nebulizer fluid containing 3 ml of water with a few MBq of different radiopharmaceuticals. Output of the radioactivity per minute of nebulization was noted for 10 min. The experiment was repeated twice for paired comparisons (n=20) between the hot and cold fluids.

### Effect of nebulizer models:

Three makes of medical nebulizers (A, B, C) were compared for drug output performance. Two were regular chambers for treating asthma, while the third was a specialized nebulizer meant for ventilation scanning. The nebulizers have been given arbitrary code names for the reason already described above. Nebulization was done with a volume of 3 ml for 10 min. Radioactivity outflow data were collected before and after nebulization.

### Effect of nebulizer chamber aging:

A fresh nebulizer system was used to generate data for drug outflow rate for different radiopharmaceuticals. To simulate ‘aging’, the system was then kept for 24 h at 0° following which hot water at 60° was kept in the chamber for 10 min. Movable parts within the chamber were moved with mild force against friction as provided by sodium chloride crystals present in the chamber. Repeat data were recorded after ‘aging’ using same protocol for different radiopharmaceuticals.

## RESULTS AND DISCUSSION

In the same experimental conditions (nebulizer system, chamber volume and solvent), different radiopharmaceuticals showed significantly different total output, drug output rate and residual drug characteristics (Tables 1, 2 and 3). Heavy particles like sulphur colloid expectedly had the least drug output, output rate and the highest residual waste. There was wide variation between nebulization potential of even soluble pharmaceuticals. Our previous work has indicated that lipophilic solutes or those lowering the surface tension might have a positive influence on nebulization rate[28]. The study confirms that the chemical nature of the solute, apart from the size in case of colloids, has a deterministic effect on nebulization rate. Consistent with the known literature, a significant percentage of drugs dissolved in water did not nebulise[32,33] indicating that the solvent aerosolizes at a much faster rate than the solutes. Similarly, irrespective of the radiopharmaceutical used, nebulization rate in the first minute was invariably higher than the mean nebulization rate and could be quantified with consistency.

Pharmaceuticals in general had a similar nebulization time for a given volume (11-14 min for 3 ml) except Tc-99m salbutamol that took significantly longer time to nebulise (21 min) (p<0.01). It also had the least mean and first-minute nebulization rates. Nebulization parameters of salbutamol are often extrapolated from the data for sodium chloride[26]. Our experience suggests that this extrapolation is inappropriate and salbutamol output is likely to be significantly over-estimated by this methodology. For this purpose, Tc-99m salbutamol data is probably more valid. We have already shown that dissolution rate of Tc-99m salbutamol correlated with that of salbutamol (by UV method) with a very high correlation coefficient (r >99.8)[30].

**Fig. 1:**
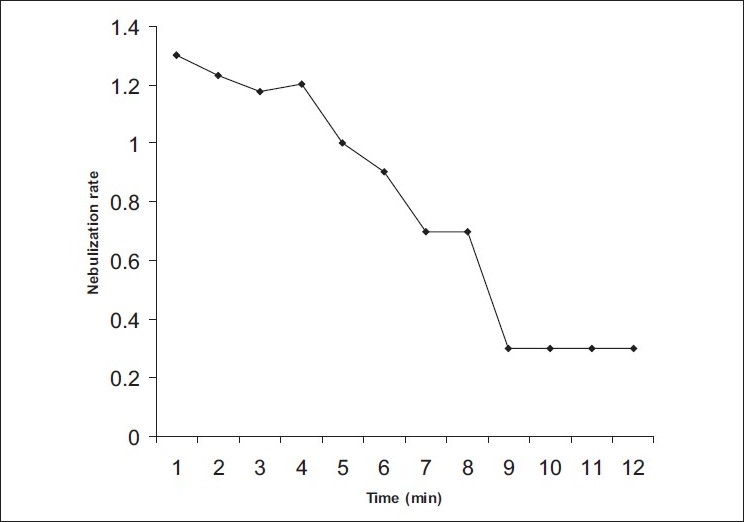
Nebulization rate per minute with Tc-99m salbutamol. Nebulization rate per minute in a series with Tc-99m Salbutamol in distilled water showing reduction in the rate with time in a roughly exponential fashion

Fig. 1 illustrates the pattern of nebulization rate with time with respect to Tc-99m salbutamol. It was seen that the rate reduces as a function of nebulization time and reaches a near-plateau long before complete evaporation of the solvent indicating that inhalation therapy is most effective at the beginning of nebulization. It is possible that increase in drug concentration in nebulization chamber with time increases viscosity that further reduces drug nebulization. The results are similar to previous extrapolations[34].

Though the specification marked for air output was same on two medical compressors (10 l/min), drug aerosol generation capacity of one was always lower compared to the other on all 18 occasions irrespective of the radiotracer used on six occasions each (p<0.01) (Table 4). The result confirms a higher degree of wear and tear of one machine, and strongly suggests that radiometric method can be used for instant quality control and standardization of air compressors used for medical purposes with the added advantage of quantification. The results are consistent with the known fact that efficiency of different systems vary with each other or with time[32,35,36].

Nebulization rate and aerosol size is dependent on the driving gas flow. Lower pressure causes formation of lesser number of aerosols with larger diameter that get trapped in baffles system of the chamber[35,36] reducing efficiency of the system further. Comparison of nebulization rates of various radiopharmaceuticals with normal and reduced air-flow is shown in Table 4. Out of 18 total readings (with 3 radiopharmaceuticals), 17 showed 10-40% lower nebulization rate than the control readings (p<0.01). Results show that reduction in aerosolization rate can be quantified using radiometry, and is independent of the drug used.

Increasing the concentration of sodium chloride in the nebulizer solution till 2% did not lead to any change in the nebulization rate (Table 5). This was rather surprising because increase in solute concentration was expected to reduce the nebulization rate, probably due to rise in viscosity[13]. It is possible that low concentration of solute causes either none or insignificant changes in the output flow rate. The results indicate that rate of drug nebulization in normal saline is equivalent to that of drug solution in distilled water in medical nebulizers.

**TABLE 4:** EFFECT ON DRUG OUTPUT BY VARIATION IN AIRFLOW

	Medical air compressor 1		Medical air compressor 2	
		
	Direct	With air resistance*	Direct	With air resistance*
Tc-99m Salbutamol	0.8±0.5%	0.6±0.3%	1.6±0.4%	1.2±0.3%
Tc-99m DTPA	2.6±0.6%	1.7±0.8%	3.7±0.7%	2.1±0.3%
Tc-99m Pertechnetate	1.9 ±0.3%	1.2±0.3%	2.5±0.5%	1.9±0.4%

* p-value less than 0.01 for all categories individually and combined. Effect on drug output by variation in airflow in different nebulizer machines (n=10 each). Comparison is made between nebulization per minute (mean ± %/min)

**TABLE 5:** EFFECT OF SODIUM CHLORIDE CONTENT ON DRUG OUTPUT AND NEBULIZATION RATE

	DW Output/rate (%)	0.45% saline Output/rate (%)	0.9% saline Output/rate (%)	2% saline Output/rate (%)
Tc-99m Salbutamol	1.2±0.3	1.4±0.5	0.9±0.2	1.4±0.2
Tc-99m DTPA	2.9±0.7	2.2±0.8	2.8±0.6	2.8±0.4
Tc-99m Pertechnetate	2.0±0.5	2.1±0.7	2.7±0.4	2.4±0.4

Effect of sodium chloride content on drug output and nebulization rate of different radiopharmaceuticals (n=30)

Ethyl alcohol and Tween-80, a strong detergent, are known to reduce surface tension. It was assumed that incremental ethyl alcohol should reduce the surface tension in proportion to its concentration, while Tween-80 will exert a more powerful surfactant action. Incremental ethyl alcohol significantly reduced the nebulization time and increased nebulization rate, but not proportionately (Table 6). The maximal increase in rate of nebulization (for 1^st^ min of nebulization) was 30-70% higher than in distilled water (or saline) while nebulization time reduced by 20-50%. Residual radioactivity was not much affected. In addition, the chamber became much colder with nebulization using alcohol, which is expected and broke in a few instances. Addition of Tween-80 resulted in frothiness upon nebulization and invariable reduction in nebulization rate. The output rate fell dramatically, and practically no nebulization or drug output occurred from the chamber even after 20 min of nebulization with 20% Tween-80 (Table 6).

**TABLE 6:** EFFECT OF ETHYL ALCOHOL AND TWEEN-80 ON NEBULIZATION

Output rate/min	Ethyl Alcohol					Tween-80		
		
	DW	5%	10%	20%	30%	0.5%	5%	20%
Tc-99m Salbutamol	1.8	2.0	2.0	3.1	2.8	2.0	2.2	Nil
Tc-99m DTPA	5.6	5.8	8.6	8.0	7.2	6.8	5.0	Nil
Tc-99m pertechnetate	5.0	5.0	6.5	8.3	7.6	5.0	3.8	Nil
Tc-99m Sulphur colloid	2.9	1.9	2.2	3.3	5.5	3.8	1.8	Nil
Tc-99m GHA	5.2	5.3	7.1	6.5	7.9	2.0	2.1	Nil

Effect of ethyl alcohol (5-30%) and Tween-80 on nebulization (n=10-30 each). Readings are nebulization rates for first minute with 3 ml volume

Viscosity of the solution is known to reduce nebulization output[13] but the effect of surface tension is more controversial with different groups reporting positive or negative effects of different quantum[37,38]. We explain this discrepancy and our observations in the following manner. Lowering of surface tension causes increase in nebulization rate but particles with larger diameter are produced. The baffles trap larger particles, reducing overall throughput. Thus, two opposing processes are operating simultaneously on nebulization rate. Till the time there is no significant trapping by baffles, lowering of surface tension shall result in a higher throughput, followed by reduction in nebulization rate as the trapping by baffles becomes more and more significant. Using the same air compressor and nebulization chamber, we were able to demonstrate this effect irrespective of the radiotracer used. Different nebulizer systems have a different cut off for trapping larger particles. Thus, groups working with different machines are likely to get different, and at times, opposing results. Even in our experiments, though the pattern was quite consistent and over-all differences were significant, there were gross variations in individual readings resulting in wide standard deviation.

Compared to nebulization fluid maintained at room temperature, drug output of cold fluid was significantly lower in initial part of nebulization. In the later half of nebulization, output rate tended to become similar. On the other hand, drug output of hot fluid upon nebulization was significantly higher in the initial part; but tended to become similar to the control values in the later half (Table 7). The pattern was independent of radiotracer used. The observation can be explained by the fact that reduction in temperature of chamber fluid results in lower Brownian motion and rise in viscosity, both contributing to reduction in nebulization process. Our results were consistent with known observations[13,38]. 

**TABLE 7:** EFFECT OF FLUID TEMPERATURE ON DRUG OUTPUT RATE

	Cold water (4°) Output rate (%/min)		Room temp. (28°) Output rate (%/min)		Hot water (60°) Output rate (%/min)	
			
	1^st^ min	6^th^ min	1^st^ min	6^th^ min	1^st^ min	6^th^ min
Tc-99m Salbutamol	0.9	1.0	1.2	1.2	1.7	1.1
Tc-99m DTPA	2.6	2.0	2.9	2.0	4.1	2.1
Tc-99m Pertechnetate	1.6	1.3	2.0	1.2	2.6	1.3

Effect of fluid temperature on drug output rate of radiopharmaceuticals (mean of 3 readings) 3 ml of water at 4° and 60° was used respectively for cold and hot water experiments

Different jet nebulizers have different output characteristics determined by design, capillary tube orifices and internal baffles. Significant and consistent difference in drug output performance of the three nebulizers used in experiments was noted. Difference in nebulization efficiency of the ‘least’ and ‘most’ efficient chamber in terms of drug output was 270% (Table 8). The results are consistent with the known facts because design of a nebulizer is probably the single most important factor influencing drug output, output rate and aerosol size properties[39]. Estimation of throughput by individual nebulization chambers is of paramount importance because a variation of upto 400% has been noticed[10,12]. This gets translated into variable and unpredictable clinical response rate in respiratory conditions like asthma and obstructive airway diseases. It also acts as an outright impediment in using inhalation as the route of delivery of systemically acting drugs negating several advantages of this route of drug delivery.

**TABLE 8:** COMPARISON OF DRUG OUTPUT AND MEAN NEBULIZATION RATE OF THREE DIFFERENT NEBULIZER CHAMBER MAKES (TOTAL OUTPUT AND MEAN NEBULIZATION RATE /MIN)

	DW Output/rate	0.45% saline Output/rate	0.9% saline Output/rate
Tc-99mSulphur Colloid	28.6%, 3.3%/min	52.9%, 7.3%/min	44.04%, 5.6%/min
Tc-99m Pertechnetate	35.0%, 3.1%/min	64.0%, 7.3%/min	54.3%, 7.0 %/min
Tc-99m DTPA	37.1%, 3.3%/min	69.0%, 7.0%/min	60.8%, 6.2%/min

Comparison of drug output and mean nebulization rate of three different nebulizer chamber makes (n=10) (Total output & mean nebulization rate /min)

Table 9 clearly shows that drug output performance and nebulization rate reduced definitively with time. With a more frequent usage and poor upkeep, efficiency of commonly available commercial plastic-body nebulizers may drop significantly and unknowingly within a few days irrespective of the drug used. Deterioration of the order of 50% suggests that nebulizer aging is also a major determining factor in nebulization therapy. Our results indicate that metal-body nebulizers may be a better option for consistent performance instead of the more popular disposable chambers. Ryan *et al.*[40] and Markus *et al.*[41] have previously showed that chamber aging may have a deleterious effect on the chamber efficiency because of reduced air flow and increased droplet size.

**TABLE 9:** COMPARISON OF DRUG OUTPUT/MEAN NEBULISATION RATE OF RADIOTRACERS IN OLD AND NEW NEBULISATION CHAMBER

	New Chamber Output/rate (%/min)	Simulated Old chamber Output/rate (%/min)
Tc-99m Salbutamol	2.0	0.9
Tc-99m DTPA	6.5	2.8
Tc-99m Pertechnetate	5.0	2.0

Comparison of drug output (n=6-10 each) or mean nebulization rate (n=10 each) of radiotracers in old and new nebulization chamber. Nebulization rate for only the first minute was considered for comparison with radiopharmaceutical in 3 ml distilled water.

We understand that the current science to evaluate jet nebulizer performance, particularly drug output parameters, is imprecise and no universally agreed methodology exists to quantify the influence of a number of known determining factors mentioned above. This has resulted in commercial presence of a number of jet nebulizers giving unknown and significantly diverse dosage of nebulised drug resulting in uneven and unpredictable clinical response. Evaluation and specification of drug delivery performance of individual compressor-nebulizer systems is necessary for all given drugs, both for medical and regulatory purposes. Through this study, we introduce Tc-99m based radiometry as a viable method for the purpose, based on preciseness and sensitivity associated with radio-immuno assay, the other more reputed in vitro use of radiometry. We used Tc-99m because of its many advantages, namely, its ability to radiolabel a large number of representative molecules including many drugs, its ability to provide scintigraphy images of the nebulizer system if needed, and easy availability. We demonstrated the utility of radiometry in documenting and quantifying the performance of jet nebulizers and in determining the effect of various factors in influencing their performance, sometimes drastically. Based on ubiquitous presence of nuclear medicine centers round the globe, we conclude that Tc-99m based radiometry methodology is a viable new technique to evaluate and probably certify drug-delivery related performance parameters and quality control of jet nebulizers.
